# Supplementary material for: Functional Characterization of the Wheat Macrophage Migration Inhibitory Factor TaMIF1 in Wheat-Stripe Rust (Puccinia striiformis) Interaction
Source: Biology (Basel). 2021 Sep 7;10(9):878. doi: 10.3390/biology10090878 (PMC8470491; doi:10.3390/biology10090878)
Supplement: Supplementary file 1 [file biology-10-00878-s001.zip › Supporting information.pdf]

# Supplementary figures and tables

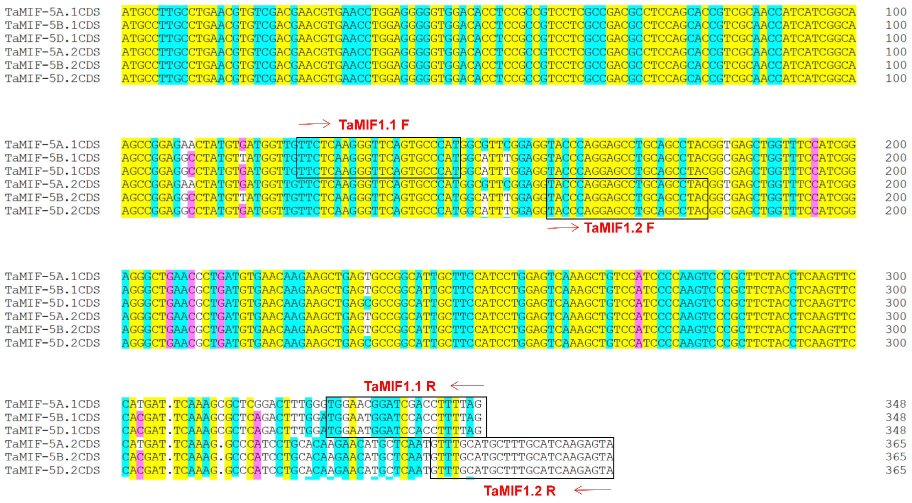


**Figure S1. Sequence alignment of TaMIF coding sequences (cds) on chromasome 5 with two transcripts.** Sequences generating identical nucleotide sequences are colored in yellow, those yielding highly similar (>75%) sequences are shown in blue, and those yielding similar (>50%) residues are shown in green. The primers used for RT-qPCR are indicated in red boxes.

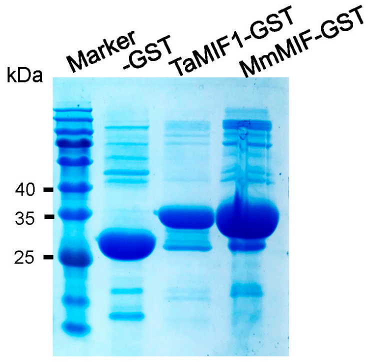


**Figure S2. Pure proteins analysis through SDS-PAGE.**

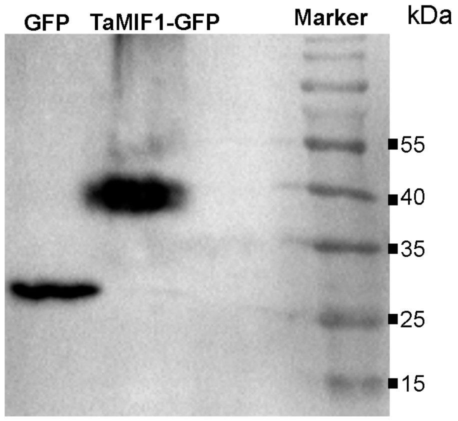


**Figure S3. The Western blot of GFP and the TaMIF1-GFP fusion protein with GFP antibodies.**

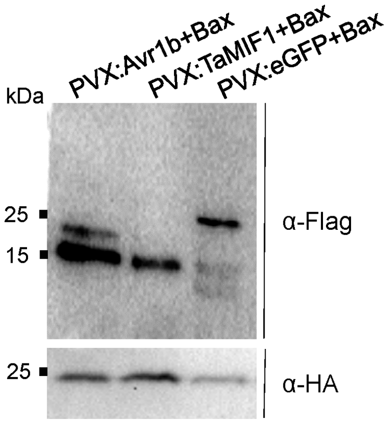


**Figure S4. Western blot analysis of protein expressions in *N. benthamiana*.** Avr1b, TaMIF1 and eGFP were detected with an Flag-antibody. Bax was detected with an HA-antibody.

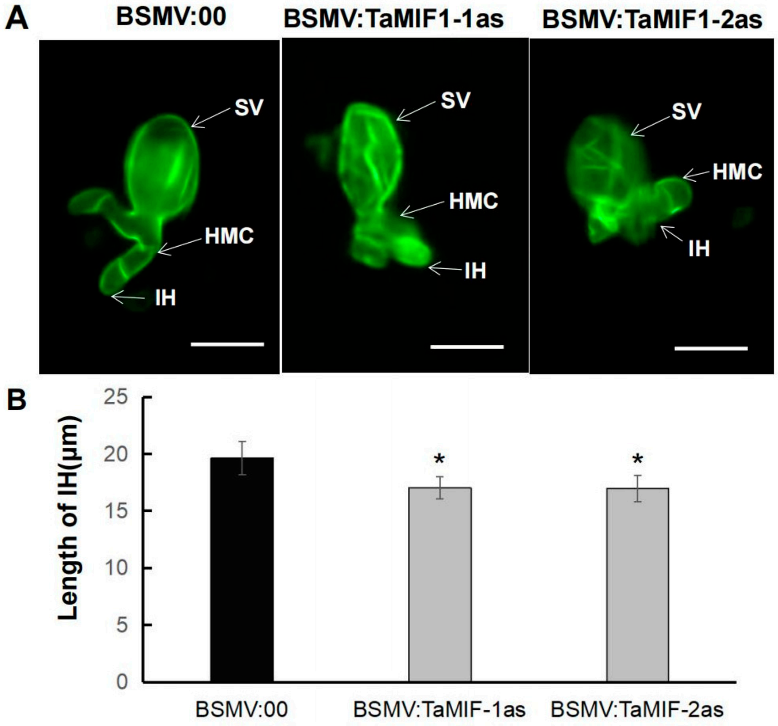


**Figure S5. Fungal growth of CYR31 in wheat leaves inoculated with BSMV:00 or BSMV:TaMIF1 at 24 hpi.** (A) After staining with wheatgerm agglutinin (WGA), fungal structure was examined under a fluorescence microscope. Bars=20 μm. (B) Length of Infective hypha was measured using DP-BSW software. The statistical results were obtained from 50 infection sites and values represent mean±SE of three independent replicates. Differences were assessed using Student's t-tests. Asterisks indicate $P < 0.05$. SV, substomatal vesicle; IH, infection hypha; HMC, haustorial mother cell.

**Table S1 Primers used in this study.**

| Primer name | Primer 5'-3' | Purpose |
|---|---|---|
| TaEF-qRT-F | TGGTGTCATCAAGCCTGGTATGGT | The endogenous reference for normalization in qRT-PCR |

| | | |
|---|---|---|
| TaEF-qRT-R | ACTCATGGTGCATCTCAACGGACT | The endogenous reference for normalization in qRT-PCR |
| TaMIF1.1-qRT-F | TTCTCAAGGGTTCAGTGCCCAT | qRT-PCR for the expression patterns of TaMIF1.1 |
| TaMIF1.1-qRT-R | CTAAAAGGTGGATCCATTCCA | qRT-PCR for the expression patterns of TaMIF1.1 |
| TaMIF1.2-qRT-F | TACCCAGGAGCCTGCAGCCTAC | qRT-PCR for the expression patterns of TaMIF1.2 |
| TaMIF1.2-qRT-R | TACTCTTGATGCAAAGCATGCAAAC | qRT-PCR for the expression patterns of TaMIF1.2 |
| TaMIF1-PTF-F | ACGCGTCGACATGCCTTGCCTGAACGTGTC | Clone TaMIF1 to pTF486 for subcellular localization |
| TaMIF1-PTF-R | CATGGGATCCAAAGGTGGATCCATTCCATCC | Clone TaMIF1 to pTF486 for subcellular localization |
| TaMIF1-4T-F | CCGCGTGGATCCCCGGAATTCATGCCTTGCCTGAACGTGTC | Clone TaMIF1 to pGex-4T for prokaryotic expression |
| TaMIF1-4T-R | ATGCGGCCGCTCGAGTCGACCCTAAAAGGTGGATCCATTCCATC | Clone TaMIF1 to pGex-4T for prokaryotic expression |
| MmMIF-4T-F | CCGCGTGGATCCCCGGAATTCATGCCTATGTTCATCGTGAAC | Clone MmMIF to pGex-4T for prokaryotic expression |
| MmMIF-4T-R | ATGCGGCCGCTCGAGTCGACCTCAAGCGAAGGTGGAACC | Clone MmMIF to pGex-4T for prokaryotic expression |
| TaMIF1-PVX-F | GGAATTCCATATGATGCCTTGCCTGAACGTGT | Clone TaMIF1 to PREP-3X for yeast overexpression |
| TaMIF1-PVX-R | CCGGAATTCCTAAAAGGTGGATCCATTCCAT | Clone TaMIF1 to PREP-3X for yeast overexpression |
| TaMIF1-PVX-F | GTACCCGGGATGCCTTGCCTGAACGTGT | Clone TaMIF1 to PVX for expression in *N. benthamiana* |
| TaMIF1-PVX-R | CATGTCGACCTAAAAGGTGGATCCATTCCAT | Clone TaMIF1 to PVX for expression in *N. benthamiana* |
| eGFP-PVX-F: | GTACCCGGGATGGTGAGCAAGGGCGA | Clone eGFP to PVX for expression in *N. benthamiana* |
| eGFP-PVX-R: | CATGTCGACTTACTTGTACAGCTCGTCCATG | Clone eGFP to PVX for expression in *N. benthamiana* |
| Avr1b-PVX-F: | GTACCCGGGATGCGTCTATCTTTTGTGCTT | Clone Avr1b to PVX for expression in *N. benthamiana* |
| Avr1b-PVX-R | CATGTCGACCAGCTCTGATACAGGTGAAAG | Clone Avr1b to PVX for expression in *N. benthamiana* |
| BAX-PVX-F | GTACCCGGGATGGACGGGTCCGGGGA | Clone BAX to PVX for expression in *N. benthamiana* |
| BAX-PVX-R | ATAAGAATGCGGCCGCGCCCATCTTCTTCCAGATGGTG | Clone BAX to PVX for expression in *N. benthamiana* |
| TaMIF1-γ-F1 | CCTTAATTAAGGGGAGGGGAGAAGAAG | Clone TaMIF1-1as to the γ vector for VIGS |
| TaMIF1-γ-R1 | ATAAGAATGCGGCCGCTCCAGGTTCACGTTCGTC | Clone TaMIF1-1as to the γ vector for VIGS |
| TaMIF1-γ-F2 | CCTTAATTAATTCTACCTCAAGTTCCACG | Clone TaMIF1-2as to the γ vector for VIGS |
| TaMIF1-γ-R2 | ATAAGAATGCGGCCGCATATGATGCGTCTAAAAGGT | Clone TaMIF1-2as to the γ vector for VIGS |

**Table S2 RNAi targets predicted of TaMIF1-1as and TaMIF1-2as.** The nucleic acid sequences of TaMIF1-1as and TaMIF1-2as were used for prediction with the RNAi off-target prediction tool

SI-FI 3.2 in both the genome of wheat and *Pst*. All predicted targets were listed, and they were all from wheat encoding TaMIF1.

| | ***Ta*MIF1-1as** | ***Ta*MIF1-2as** |
|---|---|---|
| **RNAi targets** | 5DL_AA1450270.1 | 5AL_AA1155140.1 |
| | 5BL_AA1337600.1 | 5BL_AA1337600.1 |
| | 5AL_AA1155140.1 | 5DL_AA1450270.1 |
